# Prolonged Ketamine Effects in *Sp4* Hypomorphic Mice: Mimicking Phenotypes of Schizophrenia

**DOI:** 10.1371/journal.pone.0066327

**Published:** 2013-06-18

**Authors:** Baohu Ji, Xin Wang, Antonio Pinto-Duarte, Minjung Kim, Sorana Caldwell, Jared W. Young, Margarita M. Behrens, Terrence J. Sejnowski, Mark A. Geyer, Xianjin Zhou

**Affiliations:** 1 Department of Psychiatry, University of California San Diego, La Jolla, California, United States of America; 2 Salk Institute for Biological Studies and Howard Hughes Medical Institute, La Jolla, California, United States of America; 3 Research Service, VA San Diego Healthcare System, San Diego, California, United States of America; 4 Institute of Pharmacology and Neurosciences, Faculty of Medicine, University of Lisbon, Lisbon, Portugal; 5 Neurosciences Unit, Institute of Molecular Medicine, University of Lisbon, Lisbon, Portugal; Chiba University Center for Forensic Mental Health, Japan

## Abstract

It has been well established that schizophrenia patients display impaired NMDA receptor (NMDAR) functions as well as exacerbation of symptoms in response to NMDAR antagonists. Abnormal NMDAR signaling presumably contributes to cognitive deficits which substantially contribute to functional disability in schizophrenia. Establishing a mouse genetic model will help investigate molecular mechanisms of hypoglutmatergic neurotransmission in schizophrenia. Here, we examined the responses of *Sp4* hypomorphic mice to NMDAR antagonists in electroencephalography and various behavioral paradigms. *Sp4* hypomorphic mice, previously reported to have reduced NMDAR1 expression and LTP deficit in hippocampal CA1, displayed increased sensitivity and prolonged responses to NMDAR antagonists. Molecular studies demonstrated reduced expression of glutamic acid decarboxylase 67 (GAD67) in both cortex and hippocampus, consistent with abnormal gamma oscillations in *Sp4* hypomorphic mice. On the other hand, human SP4 gene was reported to be deleted in schizophrenia. Several human genetic studies suggested the association of SP4 gene with schizophrenia and other psychiatric disorders. Therefore, elucidation of the Sp4 molecular pathway in *Sp4* hypomorphic mice may provide novel insights to our understanding of abnormal NMDAR signaling in schizophrenia.

## Introduction

In 1959, Luby et al [1] first reported their studies of phencyclidine (PCP, Sernyl) in both healthy people and schizophrenia patients. While healthy people treated with PCP displayed schizophrenia-like symptoms, the most striking finding was that exacerbation of the symptoms experienced by patients persisted for several weeks after PCP administration. The finding led to a suggestion that the action of PCP touched upon the fundamental aspect of the disease. Decades later, NMDA receptor genes were cloned, and PCP was found to be a noncompetitive N-methyl-D-aspartate receptor (NMDAR) antagonist [2], [3]. Ketamine, a weaker noncompetitive NMDAR antagonist than PCP, was later used to replace PCP in human studies [4]. Prolonged ketamine effects and exacerbation of symptoms were again observed in schizophrenia patients [5]–[7]. Direct evidence of NMDAR’s crucial role in the pathophysiology of schizophrenia came from the findings that patients with anti-NMDA-receptor encephalitis (anti-NMDAR1) developed schizophrenia-like symptoms including memory deficits [8], [9].

It has now been well established that schizophrenia patients have impaired NMDAR-mediated neurotransmission as well as an increased sensitivity and prolonged responses to NMDAR antagonists. Decreased expression of NMDAR1 was reported in postmortem brains of schizophrenia patients [10]–[12]. However, both *Nmdar1* hypomorphic mice and mice with *Nmdar1* conditionally knocked out in GABAergic interneurons displayed reduced, rather than increased, sensitivity to the effects of NMDAR antagonists on locomotion [13]–[15]. Therefore, it is necessary to establish a new mouse genetic model to mimicking both decreased NMDAR1 expression and increased sensitivity to NMDAR antagonists in schizophrenia.

Sp4, a member of the Sp1 family of transcription factors, recognizes GC-rich sequences readily identified in the “CpG islands” around the promoters of a variety of genes [16]. *Sp4* gene is expressed restrictively in neuronal cells [17], [18], and important for postnatal development of hippocampal dentate gyrus [19]. We previously reported that *Sp4* hypomorphic mice displayed several putative endophenotypes for schizophrenia and other psychiatric disorders [18]–[20]. Impaired NMDAR functions were indicated in *Sp4* hypomorphic mice with decreased expression of NMDAR1 proteins and LTP deficit [20]. Importantly, human SP4 gene was found to be deleted in schizophrenia (International Schizophrenia Consortium, 2008) 20,21, and its expression was decreased in the postmortem brains of bipolar patients [22]. Moreover, human SP4 gene single nucleotide polymorphisms (SNPs) were also reported to associate with bipolar disorder, schizophrenia, and major depression [23]–[26]. Together, these data demonstrated *Sp4* hypomorphic mice as a promising hypoglutamatergic model for schizophrenia. In the present work, we report that *Sp4* hypomorphic mice also exhibit prolonged responses to NMDAR antagonists germane to Luby’s striking findings in schizophrenia more than 50 years ago.

## Results

**Figure 1:**
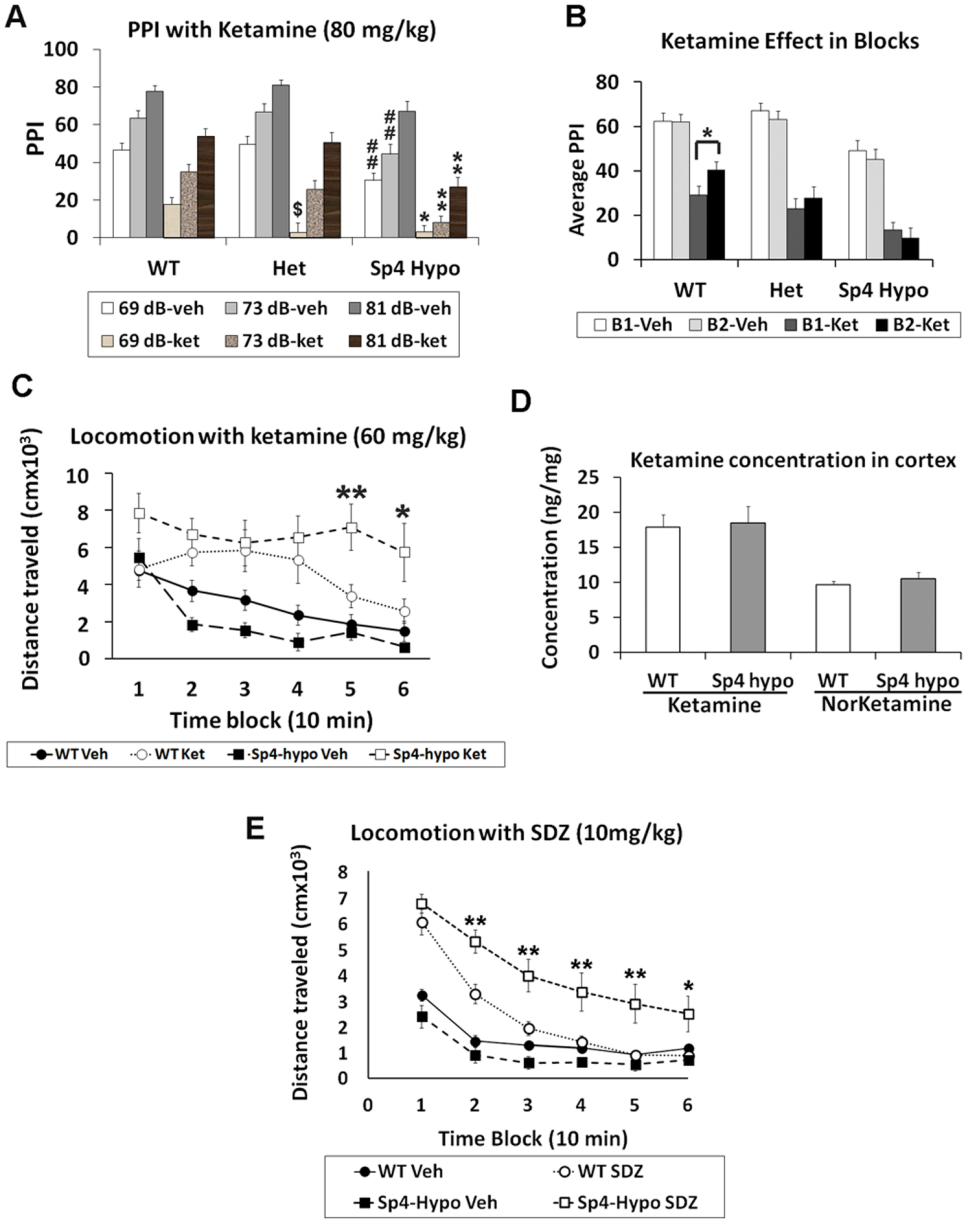
Increased sensitivity and prolonged responses to NMDAR antagonists. (**A**) There was a significant reduction of PPI in *Sp4* hypomorphic mice (n = 21) in comparison with both wildtype (n = 24) and heterozygous mice (n = 19) (## p<0.01). $: significantly lower PPI in heterozygous (p<0.05) than in wildtype mice treated with ketamine with 69 dB prepulses. Asterisks (*): significantly lower PPI in *Sp4* hypomorphic (* p<0.05; ** p<0.01) than in wildtype mice treated with ketamine. (**B**) PPI session was divided into two time blocks. Significant PPI recovery was observed during the second block (B2) in wildtype mice, but not *Sp4* hypomorphic mice, treated with ketamine. * p<0.05. (**C**) In video-track test, no significant difference between wildtype and *Sp4* hypomorphic mice treated with saline vehicle. Ketamine significantly increased total distance traveled in both groups. Significantly more locomotion was observed in *Sp4* hypomorphic mice than in wildtype mice treated with ketamine in the last 20 minutes of recording (* p<0.05; ** p<0.01). (**D**) No difference was found in the concentrations of either ketamine or norketamine in the cortex of wildtype (n = 5) and *Sp4* hypomorphic (n = 5) mice. (**E**) In BPM locomotion tests, there was a trend of lower locomotion in *Sp4* hypomorphic mice injected with saline vehicle. Administration of SDZ 220–581 increased locomotion in both genotypes, but significant more locomotion was observed in *Sp4* hypomorphic mice (n = 9) than in wildtype mice (n = 13), particularly at later blocks (* p<0.05; ** p<0.01).

F1 generation mice from two different mouse strains were generated for behavioral, electrophysiological, and molecular studies, as recommended by Banbury Conference on Genetic Background in Mice [27]. A large cohort of F1 129S/Black Swiss mice was generated to examine differential ketamine responses between wildtype and *Sp4* hypomorphic mice. Guided by previous studies on ketamine-induced PPI disruption [28], three different dosages of ketamine (60 mg/kg, 80 mg/kg, and 100 mg/kg) were tested for PPI disruption in some of the heterozygous F1 mice (data not shown). From the pilot study, a dosage of 80 mg/kg was chosen for its effective PPI disruption without sedation, and the heterozygous mice used in the pilot study were excluded for the following ketamine studies. In PPI, neither sex effect (F(1,58) = 0.38, n.s.) nor sex and ketamine interaction (F(1,58) = 0.53, n.s.) was observed between wildtype, heterozygous, and *Sp4* hypomorphic mice. Therefore, both sexes were combined for all subsequent analyses. A significant difference in PPI was observed between wildtype, heterozygous, and *Sp4* hypomorphic mice (F(2,61) = 11.69, p<0.0001) (Figure 1A). Significant gene X ketamine X prepulse intensity interaction was also observed (F(4,122) = 4.93, p<0.001). *Post-hoc* analysis revealed that *Sp4* hypomorphic mice displayed significantly lower PPI than wildtype sibling mice treated with ketamine across all three prepulse levels. No significant differences were observed in startle responses between wildtype, heterozygous, and *Sp4* hyomorphic mice, although ketamine reduced startle across all groups (Figure S1). To examine the time course of ketamine effects, the PPI session was subdivided into the first and second blocks. Time block analysis revealed a significant block X gene interaction (F(2,61) = 3.18, p<0.05) (Figure 1B). In wildtype mice treated with ketamine, a significant PPI recovery was observed in the second block from the PPI of the first block. However, no PPI recovery was observed in the second block in *Sp4* hypomorphic mice treated with ketamine, suggesting prolonged ketamine effects in the mutant mice.

Differential ketamine responses were further examined in another behavioral paradigm, video-track (VT) locomotion test. A between-subjects design was used for the VT test without prior habituation in locomotion chambers. A lower dosage of ketamine (60 mg/kg) was used to better detect gene and drug interactions. Neither sex effect nor sex and ketamine interaction was observed in locomotor activities; therefore, both sexes were combined in analysis. Between the two genotypes of mice, no significant difference in the total distance traveled was found (F(1,40) = 1.25, n.s.). However, there was a significant gene X time block interaction (F(5,100) = 2.33, p<0.05), but a trend of gene effect (F(1,20) = 3.63, p<0.1) in *Sp4* hypomorphic mice injected with saline vehicle. *Post hoc* analyses revealed significant less distance traveled at the second (p<0.05), the third (p<0.05), and a trend at the fourth (p<0.1) time blocks. Both wildtype and *Sp4* hypomorphic mice displayed habituation in total distance traveled during 60 min recording (Figure 1C). Administration of ketamine at the dosage of 60 mg/kg significantly increased the total distance traveled in both wildtype and *Sp4* hypomorphic mice (F(1,40) = 37.37, p<0.0001). As expected, a significant gene X ketamine interaction was observed (F(1,40) = 7.97, p<0.01). *Post hoc* analysis revealed that *Sp4* hypomorphic mice displayed significantly more locomotion than wildtype sibling controls at both block 5 (F(1,40) = 7.59, p<0.01) and block 6 (F(1,40) = 5.29, p<0.05), confirming prolonged ketamine responses in the mutant mice. The absence of ketamine-induced locomotion increase in the wildtype mice at the first block may be masked by hyperlocomotion because mice were not previously habituated in the chambers.

To rule out the possibility that ketamine concentrations may be different in the brains of wildtype and *Sp4* hypomorphic mice, a new cohort of mice were generated and injected with the same dosage of ketamine. The concentrations of ketamine and its metabolite norketamine were measured 15 minutes later after injection. Cortical concentrations of either ketamine or norketamine were not different between wildtype and *Sp4* hypomorphic mice (Figure 1D). Therefore, we concluded that the prolonged ketamine responses of *Sp4* hypomorphic mice resulted from the extended neural responses to the same concentration of ketamine. It was reported that ketamine could also bind to dopamine and serotonin receptors [29], although with low affinities. To examine whether the differential ketamine responses were mediated through NMDAR, not “off-target” low affinity binding receptors, a competitive NMDAR antagonist, SDZ 220–581 [30], was used in locomotion tests in a third mouse test cohort (Figure 1E). There was a trend (F(1,20) = 3.95, p<0.1) of lower locomotion in *Sp4* hypomorphic mice than wildtype sibling mice injected with saline. However, injection of SDZ 220–581, while increasing locomotion during the first two 10-min blocks in wildtype mice, generated prolonged responses in *Sp4* hypomorphic mice with a significant gene and SDZ drug interaction (F(1,20) = 21.19, p<0.001). *Post hoc* analysis revealed that *Sp4* hypomorphic mice displayed significantly more locomotion than wildtype sibling controls at all blocks except the first one. Ketamine and SDZ 220–581 are structurally different chemicals binding to different sites of NMDAR. It is unlikely that both chemicals have common “off-target” bindings to generate the same behavioral phenotypes. Therefore, we consider that alteration of NMDAR signaling in *Sp4* hypomorphic mice was responsible for the exaggerated responses to both ketamine and SDZ 220–581.

**Figure 2:**
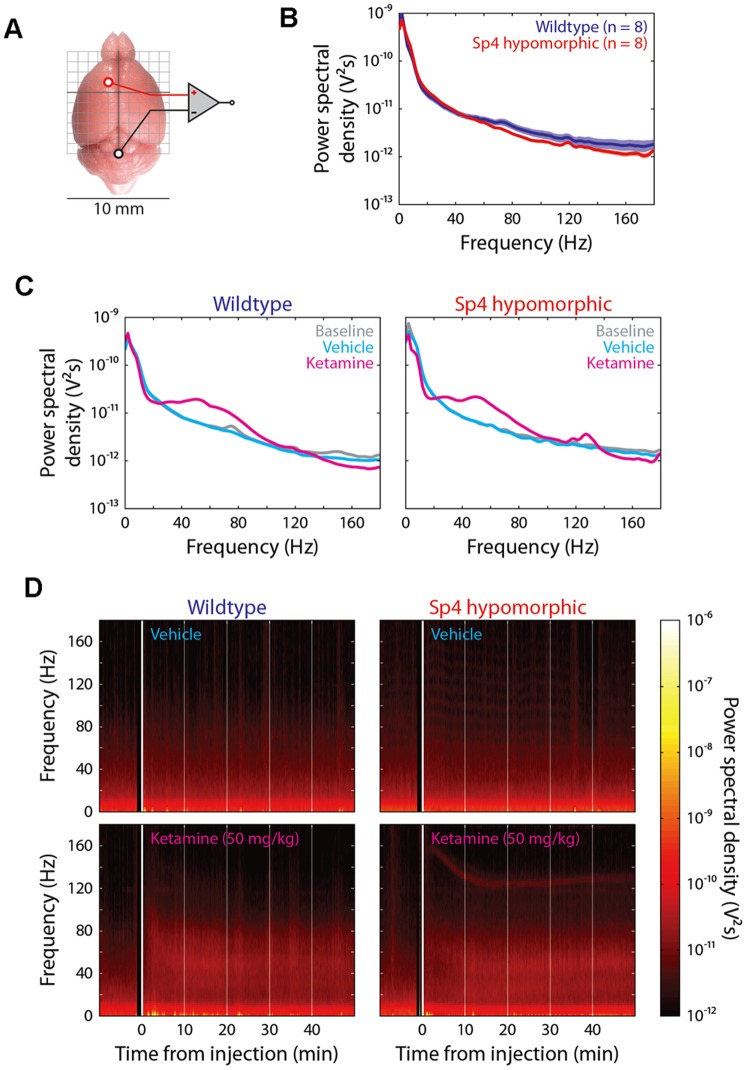
Abnormal gamma oscillations in *Sp4* hypomorphic mice. (**A**) A diagram of positions of recording and reference electrodes for epidural EEG recording. (**B**) A trend of lower EEG power of gamma oscillations (>30 Hz) was observed in *Sp4* hypomorphic (n = 8) mice than wildtype mice (n = 8). Solid lines represent population-averaged power spectra and shaded areas around the lines represent SEM. (**C**) Power spectrogram for wildtype (left) and *Sp4* hypomorphic (right) mice around the time of intraperitoneal injection of saline vehicle and ketamine (50 mg/kg). Power spectra (colored curves) integrated during a 50-minute time window after vehicle and ketamine injections were plotted in comparison to baseline spectrum (gray curve). (**D**) Illustrated data range from 10 minutes before and 50 minutes after the injection of saline and ketamine. Welch method with a Hamming window was used to estimate power spectra of 10 s, non-overlapping time bins. Black windows immediately before injections indicate a lack of data due to injection. Increase of power in low gamma (30–80 Hz) frequency range was induced by ketamine, but not vehicle, injection in both genotypes. Ketamine induced significant activity in high gamma (>80 Hz) frequency range only in the *Sp4* hypomorphic mice.

**Figure 3:**
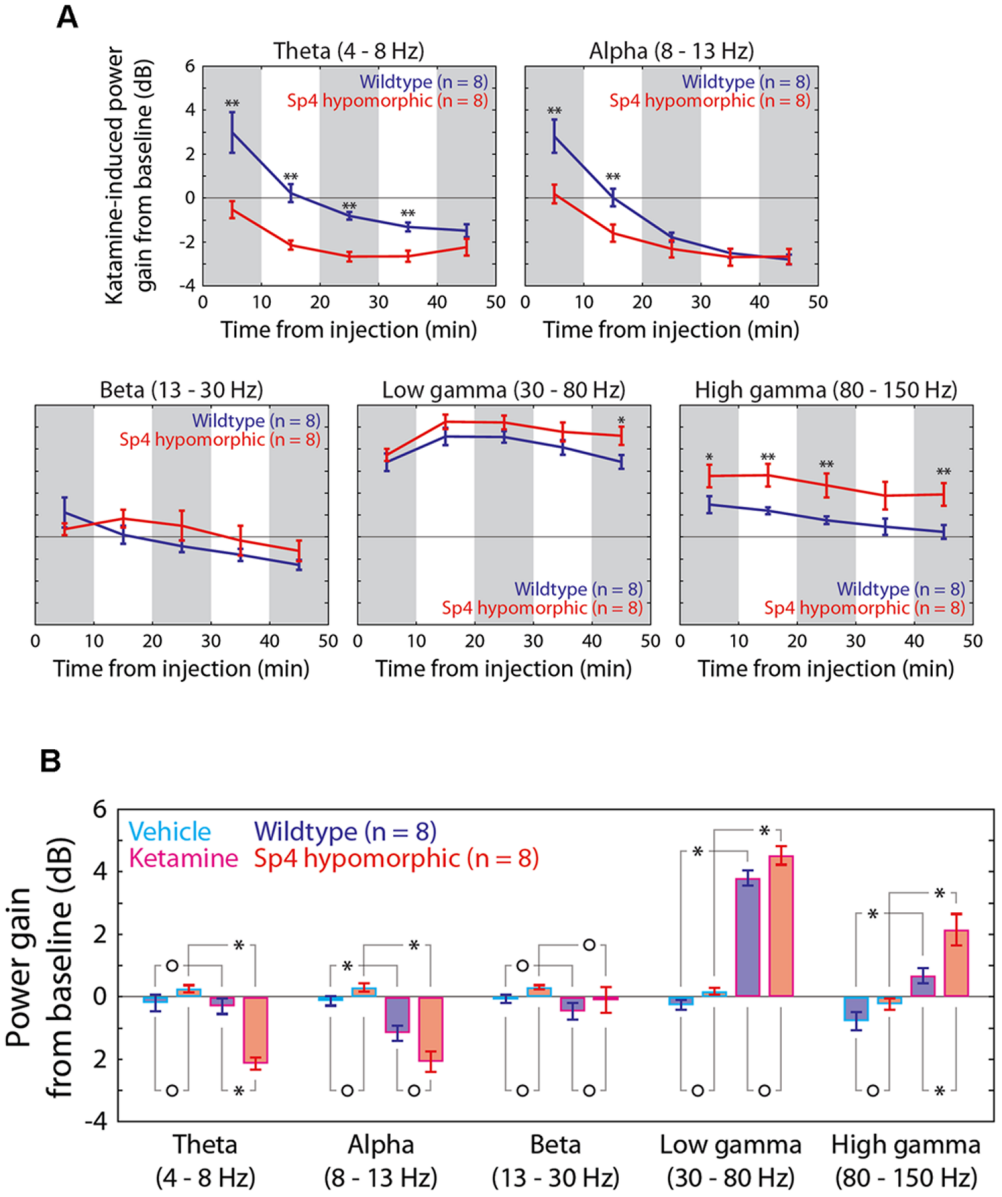
Time-course of ketamine-induced EEG power gain in various frequency ranges. (**A**) For 5 frequency ranges, i.e. theta, alpha, beta, low gamma and high gamma, EEG powers were analyzed in 10-minute bins subsequent to intraperitoneal injection of 50 mg/kg ketamine or saline vehicle. Illustrated are means and SEMs of population data for wildtype (n = 8) and *Sp4* hypomorphic (n = 8) mice. Significant results of 2-sample 2-tail Z-tests are indicated by asterisks (* p<0.05; ** p<0.01). (**B**) Vehicle- and ketamine-induced gain of power from baseline in various frequency bands for the whole dataset. Error bars represent SEM. Results of 2-sample 2-tail Z-tests were shown above and below the bar plots; Bonferroni correction for multiple hypothesis testing was used to adjust significance level (p = 0.05). Asterisks represent statistical significance and circles indicate no significance. A significant increase in high gamma, but not in low gamma, power was induced by ketamine versus by vehicle in *Sp4* hypomorphic animals. In addition, a significant reduction in theta power was caused by ketamine only in *Sp4* hypomorphic mice.

**Figure 4:**
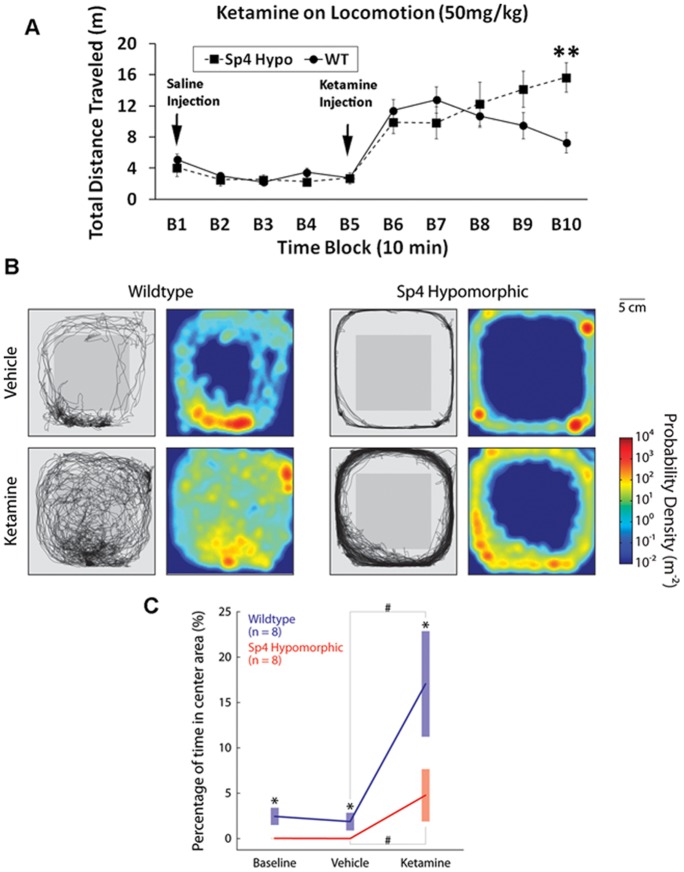
Simultaneous recording of mouse locomotion during EEG recording. (**A**) After one-hour habituation in EEG recording chamber, mice were treated with saline. Both locomotion and EEG were simultaneously recorded for 50 min after saline injection. Additional 50 minute recording was conducted after ketamine injection. *Sp4* hypomorphic (n = 8) mice displayed prolonged ketamine effects in locomotion in comparison with wildtype (n = 8) mice. (**B**) Differential locomotion patterns between wildtype (left) and *Sp4* hypomorphic (right) mice injected with saline and ketamine. (**C**) Thigmotaxis measured as the percentage of time the animal stayed in the central area of the recording chamber (gray square in B, an area 5 cm away from the edge of the chamber). Asterisks indicate significant differences between the two genotypes and pound signs indicate significant difference between saline vehicle and ketamine injections (2-sample Z-test); error bars represent SEM.

To investigate the differential ketamine responses at a neural circuit level, we generated a fourth mouse test cohort and conducted electroencephalography (EEG) and simultaneous locomotion recording (Figure 2A). We further decreased the ketamine dosage to 50 mg/kg to explore low limit of effective dosages, and examined ketamine responses after mouse habituation in chambers. Power spectral density was measured during one hour recording of individual wildtype and *Sp4* hypomorphic mouse. *Sp4* hypomorphic mice displayed a trend of lower baseline power (p<0.1) in gamma oscillations (>30 Hz) with no difference in low frequency oscillations (<30 Hz) (Figure 2B). Injection of saline vehicle did not cause any changes of baseline oscillations (Figure 2C). However, injection of ketamine significantly altered the oscillations across all frequencies in both wildtype and *Sp4* hypomorphic mice. Increase of power in low gamma (30–80 Hz) frequency range was induced by ketamine injection in both genotypes. Interestingly, a small “peak” of power spectrum was observed around 130 Hz in *Sp4* hypomorphic mice injected with ketamine. Further temporal analysis of power spectrogram illustrated a narrow band of high frequency oscillations (ranging from 130 to 160 Hz), a high gamma band induced by ketamine in all *Sp4* hypomorphic mice, but none of wildtype mice (Figure 2D). A time-course of ketamine-induced EEG power gain was examined across all frequencies of oscillations (Figure 3A). Administration of ketamine significantly increased low frequency oscillations in both theta (4–8 Hz) and alpha (8–12 Hz) bands in wildtype mice during the first ten minutes. However, no gain of EEG power was observed in *Sp4* hypomorphic mice injected with ketamine. EEG powers of both theta and alpha bands gradually decreased later after the first 10-min block in both genotypes. Beta power (13–30 Hz) was not affected by ketamine injection, and no difference was found between the two genotypes. In low gamma (30–80 Hz) range, ketamine appeared to have prolonged effects in *Sp4* hypomorphic mice to generate a significant higher power at 40–50 minutes block. In contrast to low frequency oscillations, administration of ketamine generated significantly higher powers in high gamma (80–150 Hz) bands in *Sp4* hypomorphic mice than in wildtype sibling mice. After collapsing the data of the entire 50-minute recording sessions, significantly more reduction of theta oscillations and more increase of high gamma oscillations were induced by ketamine in *Sp4* hypomorphic mice than in wildtype sibling mice (Figure 3B). During EEG recording, mouse locomotion was also recorded (Figure 4A). Mice were habituated one hour in the EEG recording chamber before injection of saline vehicle. There was no difference in total distance traveled between the two genotypes after saline injection (B1–B5). Injection of ketamine significantly increased mouse locomotion in both genotypes. As expected, *Sp4* hypomorphic mice traveled significant more distance than wildtype controls in the last 10-min block (B10). Therefore, *Sp4* hypomorphic mice displayed prolonged ketamine responses in both recording and VT chambers at different dosages of ketamine with or without prior habituation. In addition to locomotor activities, *Sp4* hypomorphic mice also displayed different moving patterns (Figure 4B). After ketamine injection, all 8 *Sp4* hypomorphic mice displayed extensive thigmotaxis moving, while most wildtype mice (7 out of 8) displayed random hyperlocomotion. The moving patterns were consistent with our previous finding that *Sp4* hypomorphic mice spent less time in the center of open field (Figure 4C) [18].

**Figure 5:**
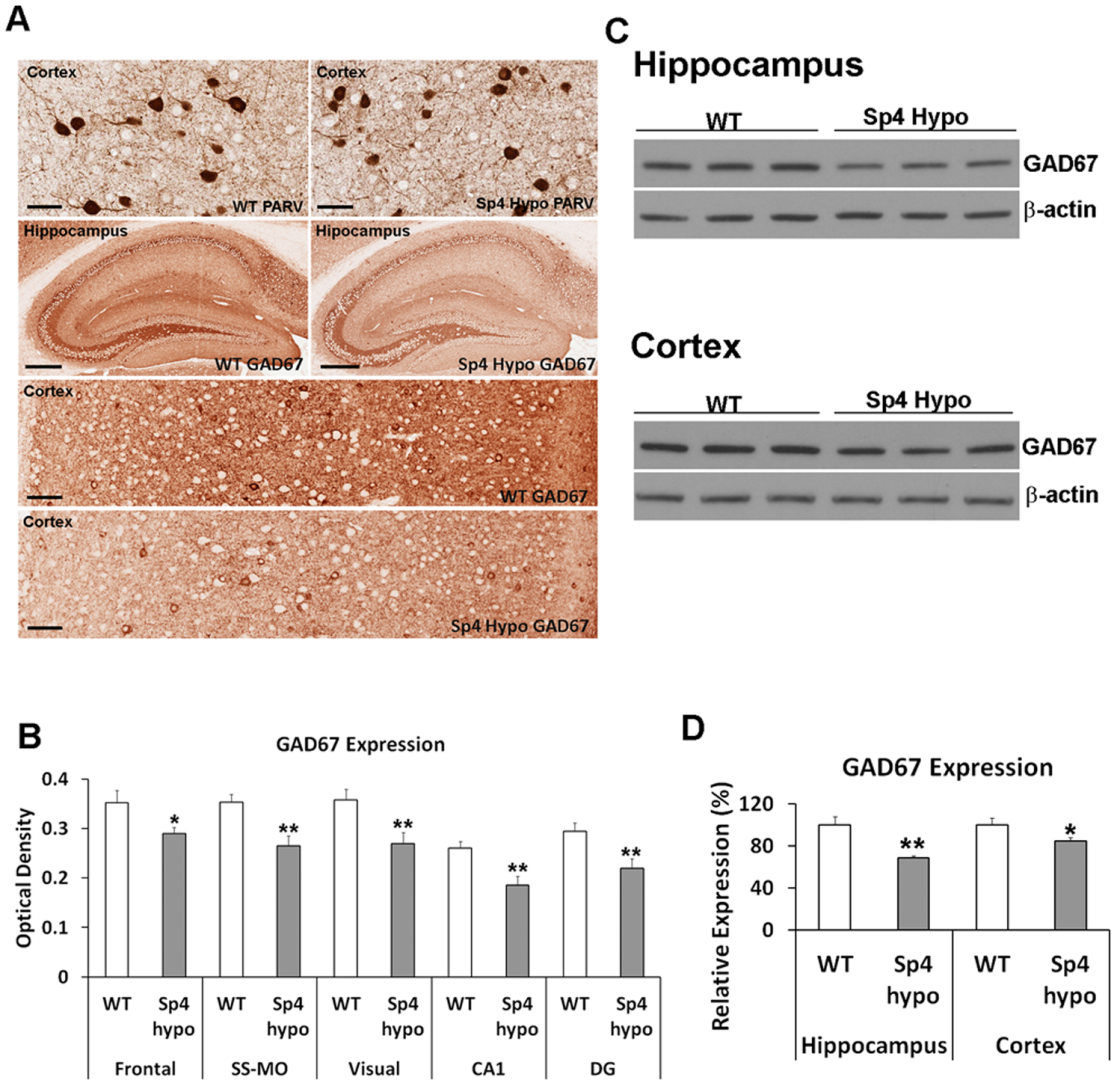
Reduced expression of GAD67 in *Sp4* hypomorphic mice. (**A**) Immunohistochemical staining of parvalbumin (PARV) and GAD67 proteins in both cortex and hippocampus of wildtype (n = 7) and *Sp4* hypomorphic (n = 7) mice. To quantify the level of protein expression, the sections were not counter-stained. The “white spots” in sections were nucleus of neuronal cells not stained by the antibodies. No difference was found in parvalbumin staining, scale bar: 25 µm. Reduced expression of GAD67 was readily detected in the hippocampus (scale bar: 250 µm) and cortex (scale bar: 60 µm) of *Sp4* hypomorphic mice. (**B**) Quantification of GAD67 protein expression in immunohistochemical staining of different brain regions (frontal cortex, somatosensory and motor cortex (SS-MO), visual cortex, hippocampal CA1, and hippocampal dentate gyrus (DG)) of wildtype (n = 7) and *Sp4* hypomorphic (n = 7) mice. Significant reduction of GAD67 staining was observed in all these regions. (**C**) Western blot analysis confirmed decreased expression of GAD67 proteins in both hippocampus and cortex of *Sp4* hypomorphic mice. Equal amount of proteins were loaded, as shown by β-actin expression. (**D**) Quantification of GAD67 protein expression in Western blot. Significant reductions of GAD67 proteins were confirmed in *Sp4* hypomorphic mice (n = 5) in comparison with wildtype sibling controls (n = 4). (* p<0.05; ** p<0.01).

Cortical GABAergic inhibitory interneurons were reported to be major targets of NMDAR antagonists [31], [32], and the activities of the interneurons generated gamma oscillations [33]. The findings of both altered ketamine responses and decreased baseline gamma oscillations in *Sp4* hypomorphic mice prompted us to examine whether the mutant mice had abnormal cortical GABAergic inhibitory interneurons, particularly parvalbumin positive interneurons [33]. We conducted immunohistochemical staining of parvalbumin in the brains of both *Sp4* hypomorphic and wildtype mice [34] (Figure 5A). The number of positive cortical parvalbumin interneurons was counted in different cortical regions. No difference was found in either the number of the interneurons or the staining intensity of parvalbumin between the two genotypes (data not shown). We next examined the expression of GAD67 proteins, a key enzyme in the synthesis of GABA neurotransmitter, in GABAergic inhibitory interneurons using immunohistochemical staining. Expression of GAD67 proteins was significantly reduced in both hippocampus and cortex of *Sp4* hypomorphic mice in comparison with those of wildtype control mice (Figure 5A, 5B). Western blot analysis confirmed the decrease of GAD67 expression in the hippocampus and cortex of *Sp4* hypomorphic mice (Figure 5C, 5D).

## Discussion

Our previous studies demonstrated that *Sp4* hypomorphic mice displayed reduced expression of NMDAR1 and LTP deficit [20]. Here, we report that *Sp4* hypomorphic mice also exhibit prolonged and exaggerated responses in various behavioral paradigms to both noncompetitive (ketamine) and competitive (SDZ 220–581) NMDAR antagonists. Our studies suggested that alteration of NMDAR signaling in *Sp4* hypomorphic mice were responsible for the differential ketamine responses. However, it remains to be investigated how NMDA neurotransmission is altered in *Sp4* hypomorphic mice. More electrophysiological studies are needed to understand the underlying molecular mechanisms in future. The present study concluded that *Sp4* hypomorphic mice present as a novel hypoglutamatergic genetic model to mimicking reduced NMDAR1 expression and increased sensitivity to NMDAR antagonists in schizophrenia.

An important question raised from our studies was how *Sp4* hypomorphic and *Nmdar1* hypomorphic mice displayed opposite responses to NMDAR antagonists, considering that both had reduced NMDAR1 expression [13], [20]. There are some differences between the two mutant mice. First, reduction of NMDAR1 was more subtle in *Sp4* hypomorphic mice than in *Nmdar1* hypomorphic mice. Second, NMDAR1 is not the only downstream gene affected by *Sp4* expression. Abnormal expression of other *Sp4* downstream genes may also contribute to their differential responses. In our studies, we found down-regulation of GAD67 proteins, a rate-limiting enzyme in GABA synthesis, in *Sp4* hypomorphic mice. Cortical GABAergic inhibitory interneurons were proposed to be the primary targets of NMDAR antagonists [31], [32]. Conceivably, reduced NMDAR functions in the GABAergic inhibitory interneurons of *Sp4* hypomorphic mice could alter ketamine responses in conjunction with decreased GAD67 expression. However, it was reported that conditional knockout of NMDAR1 in GABAergic inhibitory interneurons generated similar phenotypes to *Nmdar1* hypomorphic mice, reduced sensitivity to NMDAR antagonists [14], [15]. In these *Nmdar1* conditional knockout mice, expression of GAD67 was also decreased in GABAergic inhibitory interneurons. These data suggested that decreased expression of both NMDAR1 and GAD67 in GABAergic inhibitory interneurons were not sufficient to generate increased sensitivity to NMDAR antagonists. Moreover, recent studies suggested that excitatory pyramidal neurons could be the primary targets of NMDAR antagonists [35]. Regardless of the primary target neurons of NMDAR antagonists, however, it is possible that numerous downstream effectors of *Sp4* molecular pathway in different types of neuronal cells may interact to contribute to increased sensitivity and prolonged responses to ketamine in *Sp4* hypomorphic mice. Currently, we are conducting genetic rescue experiments to reverse behavioral and molecular abnormalities by breeding *Sp4* hypomorphic mice with *Emx1-Cre* and *Dlx6a-Cre* mouse strains to restore Sp4 expression in excitatory and GABAergic inhibitory neurons respectively. The genetic rescue experiments will evaluate the contribution of Sp4 molecular pathway in each type of neuronal cells to specific mouse phenotypes. After the dissection of behavioral phenotypes at the cellular level, molecular studies can be conducted to investigate underlying mechanisms in the right cellular context.

During EEG recording, we observed a band of high gamma oscillations (130–160 Hz) induced by ketamine only in *Sp4* hypomorphic mice. Similar narrow-band high gamma oscillations have been reported to be induced in rodents injected with increasing dosages of ketamine [36], [37]. In our current studies, the ketamine dosage used was not sufficient to induce high gamma oscillations in wildtype mice, but adequate to do so in *Sp4* hypomorphic mice, likely due to their increased sensitivity. There was no difference in locomotion between the two genotypes during the first three 10-min time blocks, ruling out the association of high gamma oscillations with locomotion. It will be interesting to know whether high gamma oscillations are associated with thigmotaxis moving pattern in *Sp4* hypomorphic mice, since high gamma oscillation was suggested to associate with psychotic symptoms [37]. Several different mechanisms have been proposed for the generation of the high gamma oscillation [38], [39]. It is unknown whether high gamma oscillations may be an intermediate phenotype of the exaggerated ketamine responses or simply a consequence of increased sensitivity of *Sp4* hypomorphic mice to ketamine.

Decreased expression of GAD67 proteins in cortical inhibitory interneurons is one of the most consistent findings in postmortem brains of schizophrenia patients 40–42. It has been suggested that GABAergic inhibitory interneurons and excitatory pyramidal neurons were responsible for the generation of gamma oscillations (>30 Hz) and lower frequency oscillations, respectively [33]. In *Sp4* hypomorphic mice, reduced expression of GAD67 proteins might contribute to the lower power of baseline gamma oscillations. Excessive release of glutamate has been reported in microdialysis studies of rodent brains after injection of NMDAR antagonists [43], [44]. Conceivably, excessive glutamate could generate initial EEG power gain (first ten-minute) of both theta and alpha oscillations via activation of excitatory pyramidal neurons in the wildtype mice. However, we did not observe any initial gain of EEG power in *Sp4* hypomorphic mice. We speculated that both theta and alpha low frequency oscillations could be dampened by reduced NMDAR functions in the excitatory pyramidal neurons in *Sp4* hypomorphic mice. Administration of ketamine overall decreased the power of low frequency theta oscillations and increased the power of high frequency gamma oscillations, which is consistent with previous reports [45]. However, such effects were exaggerated in *Sp4* hypomorphic mice.

Our studies suggested that Sp4 molecular pathway plays an important role in the modulation of ketamine sensitivity. Recently, ketamine has been demonstrated as one of most effective antidepressants in the initial treatment of depression [46]. Interestingly, human SP4 gene has been found to be one of the leading candidate genes associated with major depression [24], [25]. It is not uncommon to find the same susceptibility gene for different psychiatric disorders, as exemplified by the DISC1 translocation in a Scottish schizophrenia family [47]–[49]. Human SP4 gene was found to be deleted in schizophrenia, and its SNPs have been reported to associate with bipolar, schizophrenia, and major depression. Functional SNP variants in human SP4 gene could affect SP4 expression to alter ketamine responses. Identification of these SP4 SNPs may help predict ketamine efficacy in treatment of depression.

## Materials and Methods

### Mouse Strains and Breeding

The *Sp4* hypomorphic mice were generated as previously described [18] and maintained as *Sp4* heterozygous in 129S and Black Swiss backgrounds respectively. The *Sp4* 129S strain has been on pure 129S background since the germline transmission of the targeted *Sp4* allele, and backcrossed a few times with 129S mice to prevent potential genetic drift in the last ten years. The *Sp4* Black Swiss strain was backcrossed initially at least ten generations, and continues to be backcrossed with Black Swiss mice every other year to prevent potential genetic drift. The *Sp4* test cohort was generated by breeding the heterozygous *Sp4* mice between the two mouse strains. Therefore, all test mice were the F1 generation mice with the same genetic background. Mice were housed in a climate-controlled animal colony with a reversed day/night cycle. Food (Harlan Teklab, Madison, WI) and water were available *ad libitum*, except during behavioral testing. All behavioral testing procedures were approved by the UCSD and Salk Animal Care and Use Committee (permit number: A3033-01) prior to the onset of the experiments. Mice were maintained in American Association for Accreditation of Laboratory Animal Care approved animal facilities at UCSD and local Veteran’s Administration Hospital. These facilities meet all Federal and State requirements for animal care.

### Mouse Test Cohorts

Five different cohorts of F1 generation mice between 129S and Black Swiss strains were generated for the studies. Each cohort contained comparable numbers of males and females. The age difference was less than two weeks between individual mice. All mice were about 4–7 month old when used for studies.

#### Test cohort 1

The test cohort, consisting of 24 wildtype, 38 heterozygous, and 21 *Sp4* hypomorphic (homozygous) mice, were used to examine ketamine responses in prepulse inhibition (PPI) and video-track tests. PPI tests were conducted at 4 months of age. One month later, video-track locomotion tests were conducted.

#### Test cohort 2

The test cohort contained 5 wildtype and 5 *Sp4* hypomorphic mice, and was used for studying ketamine metabolism *in vivo*.

#### Test cohort 3

The test cohort contained 13 wildtype and 9 *Sp4* hypomorphic mice, and was used for locomotion studies in the behavioral pattern monitor (BPM) with SDZ 220–581 (10 mg/kg), a competitive NMDAR antagonist.

#### Test cohort 4

The test cohort contained 8 wildtype and 8 *Sp4* hypomorphic mice, and was used for simultaneous EEG recording and locomotion studies with injection of ketamine (50 mg/kg) or saline vehicle.

#### Test cohort 5

The test cohort contained 11 wildtype and 12 *Sp4* hypomorphic mice. Seven wildtype and 7 *Sp4* hypomorphic mice were used for immunohistochemical analysis of parvalbumin and GAD67 protein expression. Four wildtype and 5 *Sp4* hypomorphic mice were used for Western blot analysis of GAD67 protein expression.

### Ketamine and SDZ220-581

Ketamine and SDZ 220–581 were dissolved in saline and administered i.p. at a volume of 5 ml/kg immediately prior to the start of the test session [28]. The doses of ketamine and SDZ 220–581 were determined by pilot studies on some heterozygous mice that were not included in further experiments. A within-subjects crossover design was used for drug studies, with 1 week between drug treatments.

### Prepulse Inhibition

Startle reactivity and PPI were measured with startle chambers (SR-LAB, San Diego Instruments, San Diego, CA) as described [23]. All PPI test sessions consisted of startle trials (PULSE-ALONE), prepulse trials (PREPULSE+PULSE), and no-stimulus trials (NOSTIM). The PULSE-ALONE trial consisted of a 40-ms 120-dB pulse of broad-band noise. PREPULSE+PULSE trials consisted of a 20-ms noise prepulse, 80 ms delay, then a 40-ms 120-dB startle pulse (100 ms onset to onset). The acoustic prepulse intensities were 69, 73, and 81 dB (ie 4, 8, and 16 dB above the 65-dB background noise). The NOSTIM trial consisted of background noise only. The test session began and ended with five presentations of the PULSE-ALONE trial; in between, each acoustic or NOSTIM trial type was presented 10 times in a pseudo-random order. There was an average of 15 s (range: 12–30 s) between trials. A background noise level of 65 dB was presented for a 10-min acclimation period and continued throughout the test session. The amount of PPI was calculated as a percentage score for each acoustic prepulse trial type: % PPI = 100−{[(startle response for PREPULSE+PULSE)/(startle response for PULSE-ALONE)]×100}. For PPI block analysis, the middle PPI session was split into two blocks (B1 and B2). The PPI of the first block was calculated with the first five PREPULSE+PULSE trials and six PULSE-ALONE trials; and the PPI of the second block was calculated with the last five PREPULSE+PULSE trials and six PULSE-ALONE trials.

### Locomotion Tests

#### Video-track test (VT)

Locomotor activity was measured using the Video-Tracker (VT) system with four adjacent white plastic enclosures (41 X 41 X 34 cm^3^) at the floor level, which were surrounded by an opaque plastic curtain. Mice were placed separately into individual enclosures. Enclosures were wiped clean between each test session, to remove residual odors. A video camera, mounted 158 cm above the enclosures, generated the signal for the Polytrack digitizer (San Diego Instruments). Signals were processed to acquire the left-uppermost coordinate for each of the four animals simultaneously; these data were then stored in a PC computer for subsequent off-line processing and analysis. The position of each animal (*x*, *y*) (in pixels) was sampled with a frequency of 18.18 Hz, which was used to generate a coordinate file (*x*, *y*, *t*) consisting of the *x*-location, *y*-location, and the duration (time *t*) spent in that location. Each enclosure was further subdivided for analysis by the software into nine equally sized square regions. The spatio-temporal resolution of each recorded event was 0.32 cm × 0.32 cm × 55 ms, corresponding to a maximum speed of 25 cm/s.

#### Behavioral Pattern Monitor (BPM)

Spontaneous behavioral data were recorded using the BPM (San Diego Instruments, San Diego, CA), as described previously [50], [51]. In brief, a single chamber consists of a 30.5× 61× 38-cm area, with a Plexiglas hole board floor that was equipped with floor holes in the front, middle, and rear parts of the floor and eight wall holes (three along each side of the long walls and two holes in the front and back walls). Mice were tested during the dark phase of their light cycle. During testing, a white noise generator produced background noise at 65 dB. The measurement of transitions, center time, and spatial coefficient of variation were based on the nine divided regions of the chambers. The status of the photobeams was sampled every 0.1 sec. The session lasted 60 min. Raw data were transformed into the location of the animal (in X–Y coordinates), whether holepoking or rearing occurred (events), and the duration of each event (time). The chambers were cleaned thoroughly between testing sessions.

### Analysis of Ketamine Metabolism in vivo

At 4 months of age, mice were i.p. injected with ketamine at a dosage of 80 mg/kg. Because ketamine was known to be rapidly metabolized in mice, individual mouse brains were dissected out exactly 15 min later after ketamine injection following a brief transcardial perfusion with 1×PBS solution. The brains were then quickly cooled down in ice-cold PBS solution. Mouse cortex was further dissected out from 2 mm thickness coronal brain sections (from Bregma 1.32 mm to −0.82 mm) cut with Brain Matrix, and immediately homogenized and frozen in dry ice as recommended by NMS Labs (Willow Grove, PA). The concentration of ketamine and its metabolite norketamine was measured by NMS Labs.

### EEG Recording and Analysis

Mice at the age of 6–7 months were implanted with a recording device under isofluorane anesthesia. The implant device contained stainless steel epidural EEG electrodes (hair-pinned contacts at a depth of 0.5 mm from the surface of skull, using 1/8 mm diameter stainless steel wires) and a nylon-coated stainless steel subcutaneous EMG electrode channeled through an electrode interface board (EIB-8, Neuralynx). The recording electrode was positioned on frontal cortex (1 mm rostral and 1 mm lateral from bregma) and the reference electrode was placed 6 mm caudal from Bregma on mid-sagittal line (above cerebellum). Craniotomies of 0.25 mm diameter were drilled at stereotaxically positioned coordinates of electrodes. Three jewelry screws and dental acrylic were used to fix the implant onto the skull.

EEG recording sessions were conducted 2–7 days after surgery during the light cycle. Before each recording session, the animal was habituated to a transparent acrylic recording chamber 25 cm by 25 cm in size. The implant was connected to a pre-amplifier (HS-8, Neuralynx) with an interface cable to a slip-ring commutator (SL-88-10, Dragonfly); EEG and EMG signals were amplified and digitized (sampled at 1 kHz with 16-bit precision) by the Digital Lynx system (Neuralynx) and stored on a hard drive using Cheetah software (Neuralynx). Then each animal was recorded for a 3-hour session: (1) 1 hour of baseline recording, (2) at the end of the first hour, i.p. injection of a saline vehicle and another hour of recording, (3) at the end of the second hour, i.p. injection of 50 mg/kg ketamine and another hour of recording. During the whole recording session, a 65 dB white-noise background was played through speakers mounted on the recording chamber and sporadic white-noise “clicks” 30 dB above the background were administered. Animals were freely moving and video-recorded by a camera mounted on the ceiling of the recording chamber.

Recorded EEG signals were analyzed offline in Matlab using the Chronux toolbox. Artifacts (extreme voltage values exceeding 1 mV) were discarded and power spectra and spectrograms were generated using a multi-taper method. Position of the animal was extracted from video recordings by using the Computer Vision Systems Toolbox for Matlab.

### Immunohistochemical Analysis

Adult mice were anesthetized with carbon dioxide, and transcardially perfused with 2% PBS buffered paraformaldehyde. Mouse brains were dissected out and further fixed in 4% paraformaldehyde solution at 4°C for 24 hrs. After fixation, the brains were dehydrated and embedded in paraffin. Serial, saggittal sections of the brains were cut at 5 µm thickness, and then baked at 60°C oven for one hour to firmly attach the sections to glass slide. Immunohistochemical staining was conducted as described 19,34. Mouse monoclonal anti-GAD67 antibody (Sigma-Aldrich, G5419) and anti-parvalbumin antibody (Sigma-Aldrich, p3088) were diluted with antibody diluents (Dako, S3022) at 1∶60 K and 1∶4000, respectively, as the primary antibodies for overnight incubation at 4°C. ImmPRESS peroxidase-micropolymer conjugated horse anti-mouse IgG (Vector Labs, MP-7402) was used as the secondary antibody. Chromogenic reaction was conducted with ImmPACT NovaRED Peroxidase Substrate (Vector Labs, SK-4805). Slides were mounted with Cytoseal 60 mounting medium (Richard-Allan Scientific, 8310-16). For the quantification of parvalbumin positive cells, Image J was used for particle analysis with the parameters (particle size: 25 um-infinity; circularity: 0.3–1.00). The threshold was adjusted to suppress the background of individual slides, and counting accuracy was about 99% in overlay images. The GAD67 expression was quantified with Image J using optical density. All images were first converted into 8-bit grayscale images. Before the quantification, a calibration of optical density was conducted for analysis of all images according to the instructions (http://imagej.nih.gov/ij/docs/examples/calibration/).

### Western Blot

Proteins were extracted from mouse cortex and hippocampus, and measured with Bradford (Abs 595 nm) method using Coomassie Plus Protein Assay (Thermo Scientific, IL). After electrophoresis, proteins were transferred onto PVDF membranes which were subsequently blocked with 5% nonfat dry milk in TBST buffer (pH 7.5, 10 mM Tris-HCl, 150 mM NaCl, and 0.1% Tween 20) at room temperature for 1 hour. The membranes were incubated with the following primary antibodies at 4 °C overnight: mouse monoclonal anti-GAD67 antibody (Sigma-Aldrich, G5419) (1∶10,000); sc-25270, mouse monoclonal anti-β-Actin (1∶5,000). After three times washing, the membranes were further incubated with horseradish peroxidase (HRP)-conjugated anti-mouse IgG (1∶5,000, Cell signaling, MA) for 1.5 hour at room temperature. Quantification of proteins expression was conducted with Image J.

### Statistical Analysis

For statistical analyses, repeated measures analysis of variance (ANOVA) with genotypes as between-subjects factor and drug treatment, block and prepulse intensity as within-subjects factors were performed on the %PPI data and total distance traveled. *Post hoc* analyses were carried out using Newman-Keuls or Tukey’s test. Alpha level was set to 0.05. All statistical analyses were carried out using the BMDP statistical software (Statistical Solutions Inc., Saugus, MA). For statistical analysis of parvalbumin and GAD67, unpaired student’s *t*-test (significance level, 0.05) was used.

## Supporting Information

Figure S1 Startle and startle habituation in *Sp4* hypomorphic mice injected with ketamine. Administration of ketamine decreased startle across all groups. No significant differences were observed in startle responses between wildtype, heterozygous, and *Sp4* hyomorphic mice.
(TIF)
