# Supplementary figures and images for: Prolonged Ketamine Effects in Sp4 Hypomorphic Mice: Mimicking Phenotypes of Schizophrenia
Source: PLoS One. 2013 Jun 18;8(6):e66327. doi: 10.1371/journal.pone.0066327 (PMC3688895; doi:10.1371/journal.pone.0066327)

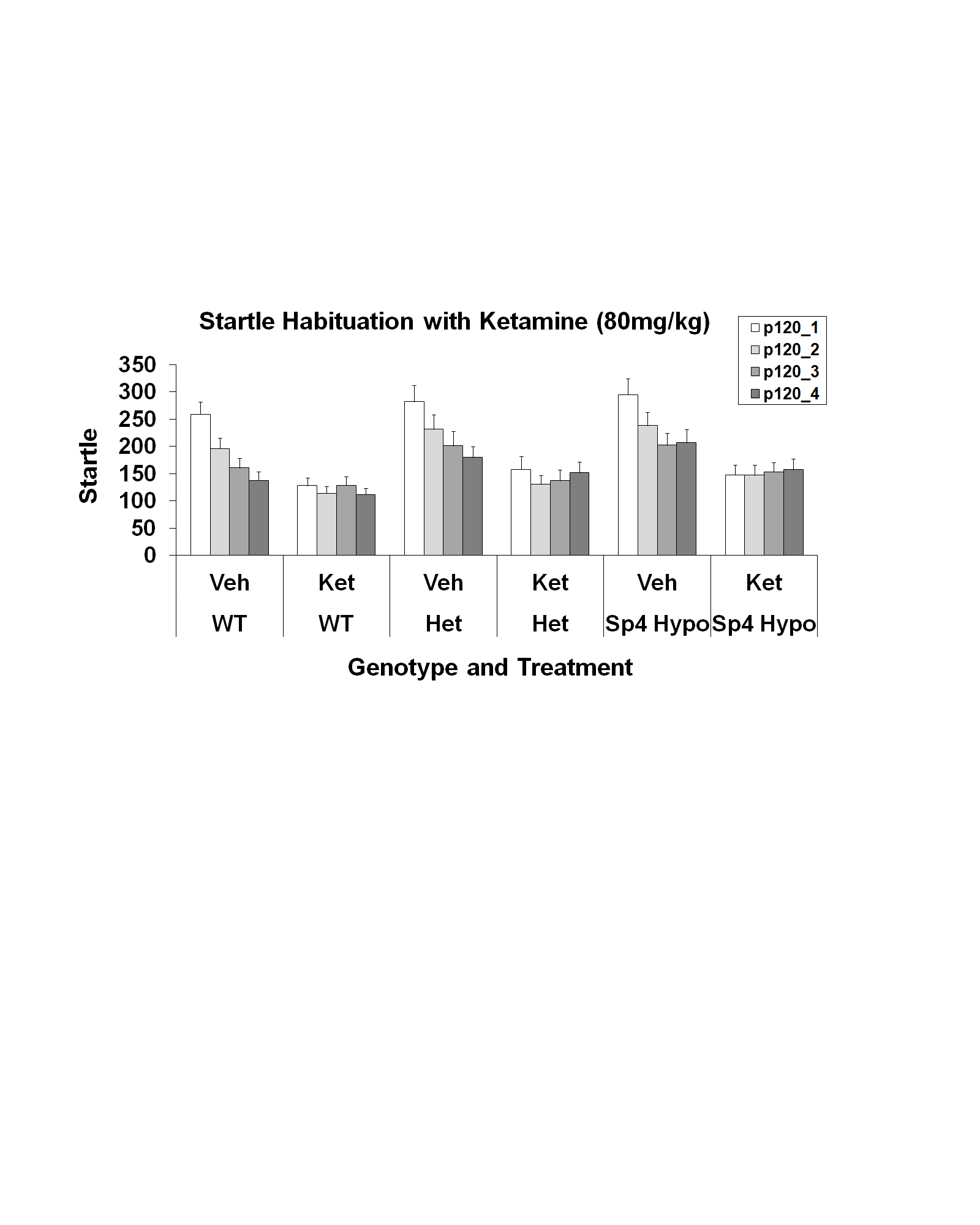

Supplement: Figure S1 — Startle and startle habituation in Sp4 hypomorphic mice injected with ketamine. Administration of ketamine decreased startle across all groups. No significant differences were observed in startle responses between wildtype, heterozygous, and Sp4 hyomorphic mice. (TIF) [file pone.0066327.s001.tif]
